# Rapid Scientific Promotion of Scientific Productions in Stem Cells According to The Indexed Papers in The ISI (web of knowledge)

**DOI:** 10.22074/cellj.2016.3750

**Published:** 2015-07-11

**Authors:** Rahim Alijani

**Affiliations:** Department of Knowledge and Information Science, Payame Noor University (PNU), Tehran, Iran

**Keywords:** Production, Scientific Integrity Review, Iranian, Stem Cells, Bibliographic Database

## Abstract

**Objective:**

In recent years emphasis has been placed on evaluation studies and the publication of scientific papers in national and international journals. In this regard the publication of scientific papers in journals in the Institute for Scientific Information (ISI) database is highly recommended. The evaluation of scientific output via articles in journals indexed in the ISI database will enable the Iranian research authorities to allocate and organize research budgets and human resources in a way that maximises efficient science production. The purpose of the present paper is to publish a general and valid view of science production in the field of stem cells.

**Materials and Methods:**

In this research, outputs in the field of stem cell research are evaluated by survey research, the method of science assessment called Scientometrics in this branch of science. A total of 1528 documents was extracted from the ISI database and analysed using descriptive statistics software in Excel.

**Results:**

The results of this research showed that 1528 papers in the stem cell field in the Web of Knowledge database were produced by Iranian researchers. The top ten Iranian researchers in this field have produced 936 of these papers, equivalent to 61.3% of the total. Among the top ten, Soleimani M. has occupied the first place with 181 papers. Regarding international scientific participation, Iranian researchers have cooperated to publish papers with researchers from 50 countries. Nearly 32% (452 papers) of the total research output in this field has been published in the top 10 journals.

**Conclusion:**

These results show that a small number of researchers have published the majority of papers in the stem cell field. International participation in this field of research unacceptably low. Such participation provides the opportunity to import modern science and international experience into Iran. This not only causes scientific growth, but also improves the research and enhances opportunities for employment and professional development. Iranian scientific outputs from stem cell research should not be limited to only a few specific journals.

## Introduction

Stem cell research is considered to be one of the most important fields in Iranian medical research, and one which has improved significantly in recent years. According to the 2014 obtained results from the Institute for Scientific Information (ISI) database, the United State of America has occupied first place in the field of stem cell research by producing 104306 scientific papers. Among countries in the region, only Turkey, which is 26^th^ in rank, and Iran, which is in 29^th^ in the world, regarding the number of scientific productions in the field of stem cells are worthy of mention. One criterion of a country’s stage of development and evolution is its research potential and scientific capacity, and one of the most important indicators of this is the number of indexed scientific papers in valid and creditable databases. Hereby, through measurement of scientific productions, it is possible to provide a view of the scientific activity status of a country. Today, these citation databases form the base of several studies that evaluate the scientific level of countries of the world using various scientometric methods.

Scientometric experts believe that each researcher in any area or field is and should be able to engage with scientometric activities so that, he/she can clearly present the current state of research to peers and officials, and thereby help them make more effective decisions (1). The first definitions of scientometrics were expressed by its founders. Scientometrics is the study of measuring and analyzing science, technology and innovation, it should be added that scientometrics is regarded as a part of social science.

The history of introducing "scientometrics" as a term refers to 1969, when a Russian scientist named Vasili Vasilevich Nalimov used the term Naukometrija in an article which means scientometrics in English. In a short time, this term was translated into English under the term of "scientometrics" (2).

Although it is several decades since scientometrics emerged internationally and a journal with this title (SCIENTOMETRICS) has been successfully published since 1978, scientometrics is almost new in Iran, although it has appeared in recent years under different heading, most frequently bibliometrics.

As it mentioned before, researchers in the field of scientometrics believe that researchers and experts in all fields should be involved with scientometrics. It is with this in mind that the present study has been conducted. Using information from ISI and other databases a number of different studies have been done, some of which are mentioned here.

A study in the same field with the title "evaluation of studies related to stem cells", which included four countries; Taiwan, Hong Kong, Singapore and Korea, based on the ISI database was conducted outside our country. Findings and results of this study showed that, although Taiwan is the first country to have done primary scientific research in this field, scientific output from South Korea has outstripped the other countries. Hong Kong and Taiwan showed a good growth too. The results of this study also showed that seven industrial countries, named Group 7 (USA, Japan, Germany, England, France, Italy and Canada), produced more than 70% of the scientific research in this field from 1981 to 2001 (3).

The other research done by Li et al. (4) under the title "bibliometric study of global tendency for performing research on the stem cells during 1991 to 2006" showed that over that period scientific productions in this field improved significantly. Scientific productions regarding stem cell research has been published in 2493 journals and 163 subject area accordingly. Most of the studies were done on homothology, ancology and cellular biology. Group 7 is also in first place regarding scientific output in other fields. According to study based on Medline, about 2695 papers from Iranian researchers were available from 1976 to 2003. Of these, 84% were written by one to three authors. Tehran Medical Science University has occupied first place among the Iranian institutes. Also 35% of printed journals in which Iranian researchers have published their papers are based in the USA, and 24% in England (5).

Another study evaluated scientific output in the field of medical science based on indexed papers in scientific journals in selected databases from 2005 to 2009. The results of this study showed that the number of publications in the field of medical science was lacking, such that during the years of the evaluation, Iranian scientific output in the field of medicine was developing (6).

An evaluation was conducted of 625 papers in sciences websites from the Medical Science of Iran University up to the end of 2007. The results of this study showed that in five case studies made on immunology, circulatory system, neuroscience, surgery and pharmacology, only three authors wrote the article individually and this fact indicated the high tendency of many writers in scientific collaboration and co-authorship. According to this research, the highest level of scientific cooperation is found in the field of immunology (7).

Poris has investigated the status of acquired immune deficiency syndrome (AIDS) scientific output in South Africa and has compared it with other countries from 1996 to 2006. The findings showed that the total number of AIDS papers that were on the ISI website during the studied period indicated a 42% increase. It includes approximately 5% of AIDS research at global level. Also the subject fields of immunology, public health and virology respectively, have obtained the highest share of AIDS research of this country. The pattern of international cooperation among the authors showed that about 24% of AIDS papers have been composed with the cooperation of American researchers. England, Canada and France, with a total of 2%, are also significant collaborators in South African research. In relation to institutions, Harvard and California University have cooperated the most with South Africa Organizations for accomplishing research projects (8).

With this introduction and due to the important role of research in all fields, this study intends to investigate the scientific output of Iranian researchers in the field of stem cells according to the ISI databases. The study will identify the strengths and weaknesses of research in this field sp practitioners and researchers can take appropriate action to remedy the weaknesses as well as further developing the strengths.

## Materials and Methods

This survey research includes all papers by Iranian researchers in the field of stem cell research that have been reviewed from 1992 to July of 2014, based on the Web of Science citation index, which is a sub database of ISI institution in Philadelphia of USA.

According to the Journal Citation Reports (JCR), more than 13000 core journal in the ISI database are indexed. For doing this research the key word "stem cell" was entered. By entering this key word, all the items such as: "stem cell" , "stem cells", "stem cell containing", "stem cell transplant" , "stem cellulose" were retrieved. Also, a general search about stem cells was done to identify pioneer countries in scientific outputs in this field as well as other countries in the region. All the records found were investigated regarding the following items: international participation, organization affiliation, the years of printing the papers, the format of papers, the name of journals that have printed the most papers, and the subjects that are studied by researchers. The found data was analyzed and evaluated Excel software. To ensure the accuracy of the data, the same searches were undertaken again in the ISI database at the end of the analyses. The result of this second searching process was exactly the same as that obtained from the first search.

## Results

**Fig.1:**
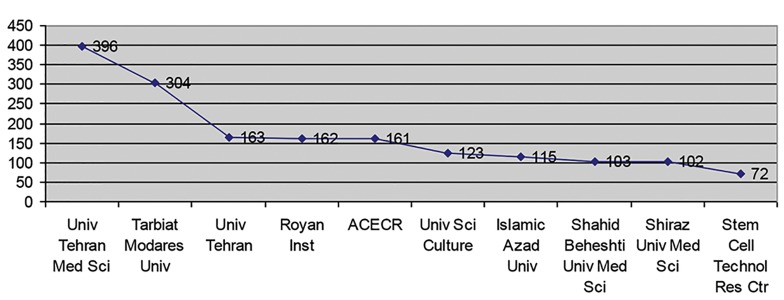
Top ten universities or top institutions.

According to the table 1 and figure 1, Tehran University of Medical Science, with 396 papers, which is equal to 25.92% of the total scientific output has occupied the first place. Tarbiat Modares University with 304 papers, Tehran University with 163 papers, and the Royan Institute with 162 papers have occupied second, third and fourth places respectively. Other universities and institutions are shown in table 1.

**Table 1:** Top ten universities or top institutions


No.	Universities or topinstitutions	Number ofpapers	Percent

1	Univ Tehran Med Sci	396	25.92
2	Tarbiat Modares Univ	304	19.90
3	Univ Tehran	163	10.67
4	Royan Inst	162	10.60
5	ACECR	161	10.54
6	Univ Sci Culture	123	8.05
7	Islamic Azad Univ	115	7.53
8	Shahid Beheshti Univ Med Sci	103	6.74
9	Shiraz Univ Med Sci	102	6.68
10	Stem Cell Technol Res Ctr	72	4.71


**Fig.2:**
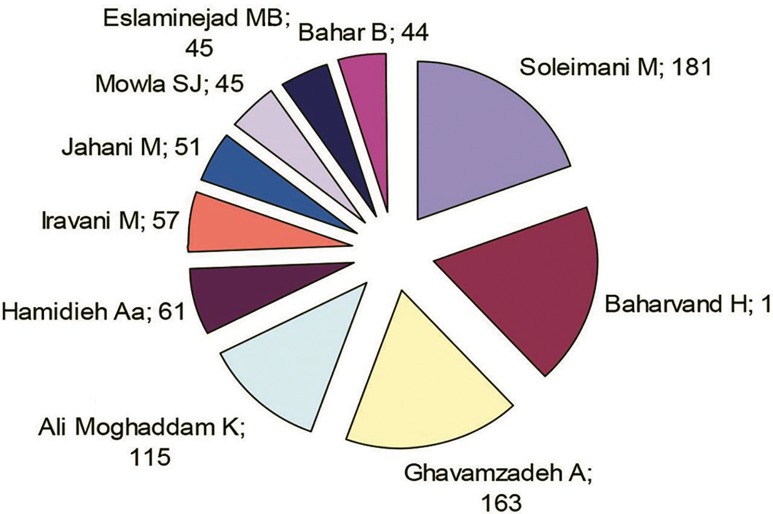
Top 10 authors.

According to table 2 and figure 2 Soleimani M, with 181 papers equal to 11.846 papers of the total scientific productions of this field is in the first place. Baharvand H, with 174 papers and Gavamzadeh A, with 163 papers are in second and third place respectively. In general, the top 10 authors all together have participated in providing 936 papers equal to 61.25% of total scientific productions of this field.

**Table 2:** Top 10 authors


No.	The name of top writers	Number	Percent

1	Soleimani M	181	11.84
2	Baharvand H	174	11.38
3	Ghavamzadeh A	163	10.66
4	Alimoghaddam K	115	7.52
5	Hamidieh Aa	61	3.99
6	Iravani M	57	3.73
7	Jahani M	51	3.33
8	Mowla SJ	45	2.94
9	Eslaminejad MB	45	2.94
10	Bahar B	44	2.88
	Total	936	61.25


According to table 3, all the scientific outputs in this field were produced in collaboration with other scientific researchers and each paper belongs to more than one author. Researchers in this field have cooperated with the researchers in the USA in producing 98 papers equal to 6.414% of total scientific output. The USA occupies the first place in this regard. Also England with 53 papers and Germany with 44 papers occupy the second and third places respectively. Researchers from Iran have cooperated with the researchers from 50 countries in producing scientific papers. These 50 countries have participated in producing 586 papers, equal to 38.35% of the total scientific output.

**Table 3:** Scientific and international participation


No.	The name of country	Number	Percent

1	Iran	1528	100
2	USA	98	6.414
3	England	53	3.469
4	Germany	44	2.88
5	Canada	44	2.88
6	Australia	39	2.552
7	France	20	1.309
8	Netherlands	19	1.243
9	Malaysia	18	1.178
10	Italy	18	1.178


According to table 4, Yakhteh (Cell J), belonging to the Royan Institute, has published 187 papers and in this regard has occupied the first place The journal Bone Marrow Transplantation, with 87 papers equal to 5.6%, is in second place and Cell Biology International with 34 papers, equal to 2.2% , is in third place. Generally, the top 10 journals shown in table 4 have published 452 papers, equal to 31.9% of the total scientific output of Iranian research in this field.

**Table 4:** The journals publishing scientific output in this field


No.	The name of top writers	Number	Percent

1	Yakhteh (Cell Journal)	187	12.24
2	Bone MarrowTransplantation	87	5.69
3	Cell Biology International	34	2.23
4	Biology Of Blood AndMarrow Transplantation	31	2.03
5	Iranian Journal Of BasicMedical Sciences	25	1.64
6	In Vitro CellularDevelopmental BiologyAnimal	23	1.51
7	Cytotherapy	23	1.51
8	Stem Cells and Development	22	1.44
9	Iranian Red CrescentMedical Journal	22	1.44
10	Clinical Biochemistry	21	1.37
	Total	452	31.9


**Fig.3:**
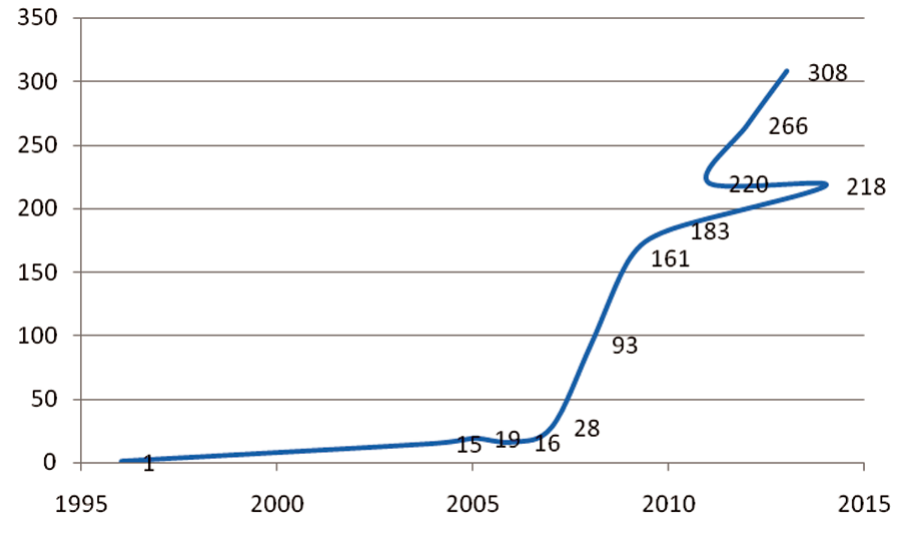
The trend in scientific output.

According to table 5 and figure 3, the trend in Iranian researchers’ scientific output in this field has been increasing and an accelerating trend in output in this field can be clearly observed. As it is shown in table 5, while in 1996 just 1 paper was indexed ISI and no papers were indexed from 1997 to 2003, progress of terms of scientific output in this area started from 2004. Although initially growth was slow, it increased steadily and then accelerated rapidly in 2013 when it reached its peak.

**Table 5:** The trend in scientific output


No.	Year	Number of papers	Percent

1	2013	308	20.16
2	2012	266	17.41
3	2011	220	14.40
4	2014	218	14.27
5	2010	183	11.98
6	2009	161	10.54
7	2008	93	6.09
8	2007	28	1.83
9	2006	16	1.05
10	2005	19	1.24
11	2004	15	0.98
12	1996	1	0.07
	Total	1528	100


According to table 6, all Iranian scientific papers in this field have been published in form of 8 types of papers among which ARTICLE with 1123 papers equal to 73.49 percent is in first place and MEETING ABSTRACT with 252 papers and REVIEW with 107 papers are in second and third places respectively.

**Table 6:** Types of papers


No.	Types of papers	Number	Percent

1	Article	1123	73.49
2	Meeting Abstract	252	16.49
3	Review	107	7
4	Proceedings Paper	20	1.31
5	Letter	12	0.79
6	Editorial Material	9	0.59
7	Book Chapter	3	0.20
8	Correction	2	0.13
	Total	1528	100


## Discussion

The results of this research showed that, overall, Iranian researchers’ have produced 1528 papers in the field of stem cell research. In terms of institutions, Tehran Medical Science University, with 396 papers equal to 25.92% of all the scientific output, is in first place. Tarbiat Modares Univ with 304 papers and Tehran University with 163 papers and the Royan Institute with 162 papers are in second, third and fourth places respectively. Overall, 320 organizations have participated in scientific output in this field. The most active researchers in this field, Solimani M, with 181 papers equal to 11.846% of the total scientific output in this field is in first place and Baharvand H, with 174 papers and Gavamzadeh A, with 163 papers are in second and third places respectively.

In general, the 10 top authors in this field, altogether have participated in producing 936 papers equal to 61.25% of the total scientific output. Regarding scientific participation all scientific productions in this field have involved co-operation (Table 3) and not one scientific paper in this field has been published or authored by one individual. Researchers have co-operated with researchers in the USA in 98 papers, equal to 6.414% of the total scientific output. Accordingly the USA is in first place with respect to cooperation. England with 53 papers and Germany with 44 papers occupy second and third places respectively. According to the findings of this part of the study, Iranian researchers have collaborated with researchers from 50 countries. These 50 countries have participated in providing 586 papers, equal to 38.35% of the total scientific output. Of the total scientific output (1528 papers), a little more than one third include international participation, Since the science of stem cells is new, it is necessary to make use of these experiences, and common research projects will be accomplished through this international participation. To facilitate this, practitioners should make appropriate decisions and suggest solutions such as providing research opportunities, giving scholarships and sending students abroad in order to encourage more international participation in this field.

Considering the journals that have printed the most scientific productions in this field, Yakhteh (Cell J) belonging to the Royan Institute, has published the most papers in this area in Iran has occupied first place in this regard. The journal of Bone Marrow Transplantation with 87 papers equal to 5.69% is in second place and Cell Biology International with 34 papers equal to 2.23% is in third place. As a whole, top the 10 journals have printed 452 papers equal to 31.9% of the total scientific output in this field. Iranian researchers' scientific papers in this area have been published in 201 journals.

Taking the presented papers format into consideration, all of the Iranian contributions to the field of stem cell research have been printed in the form of 8 types of papers among which ARTICLE with 1123 papers equal to 73.49% is in first place and MEETING ABSTRACT with 252 papers and REVIEW with 107 papers are in second and third places respectively.

## Conclusion

The contribution of Iranian publications to the field of stem cell research was evaluated according to papers cited on the ISI database. During the period covered by this research, from 1996 to 2013, Iranian contributions have followed a growing trend and rapid growth and development of scientific output in this field is clearly evident. In terms of international collaboration perhaps more could be done. Regarding the journals that have printed scientific papers in this field, the results show that the researchers of this field have limited their scientific productions only to specific journals. This case is also not desirable and by recognizing other journals of this field, this problem should be resolved.

## References

[1] Alijani R, Karami N (2008). Metrics studies: bibliometrics scientometrics informetrics webometrics.1st ed.

[2] Arunachalam S (2003). Information for research in developing countries-Information technology, a friend or foe. Int Inf Libr Rev.

[3] HO YS, Chiu CH, Tseng TM, Chiu WT (2003). Assessing stem cell research productivity. Scientometrics.

[4] Li LL, Ding G, Feng N, Wang MH, Ho YS (2009). Global stem cell research trend: Bibliometric analysis as a tool for mapping of trends from 1991 to 2006. Scientometrics.

[5] Osareh F, Marefat R (2006). Cooperation of Iranian researchers in Medline database. Rahyaft.

[6] Abdekhoda H, Ghazi MirSaeed SJ, Nourzi A (2010). Evaluation of scientific production of Iranian medical domain based on the document indexed from scientific journals in chosen databases between 2005-2009. Payavarde Salamat.

[7] Hassanzadeh HM, Gorji HA, Shokranehnanehkaran F, Valinejadi A (2009). Scientific products of Iran University of Medical Sciences’ authors with co- authorship networks in Web Of Science (WOS) database, up to 2007. Journal of Health Administration.

[8] Pouris A, Pouris A (2011). Scientometrics of a pandemic: HIV/ AIDS research in South Africa and the world. Scientometrics.

